# Supplementary material for: The Memory Abilities of the Elderly Horse
Source: Animals (Basel). 2024 Oct 25;14(21):3073. doi: 10.3390/ani14213073 (PMC11545349; doi:10.3390/ani14213073)
Supplement: Supplementary file 1 [file animals-14-03073-s001.zip › animals-3209500-supplementary.pdf]

Table S1: Times recorded by horses at T1

	Adult horses T1a	Adult horses T1b	Adult horses T1c	Senior horses T1a	Senior horses T1b	Senior horses T1c
1.	180	180	48.03	180	180	48.03
2.	6.55	33.08	8.43	6.55	33.08	8.43
3.	11.34	8.69	24.51	11.34	8.69	24.51
4.	4.64	15.01	2.17	4.64	15.01	2.17
5.	99.31	180	80.61	99.31	180	80.61
6.	11.09	28.33	9.9	11.09	28.33	9.9
7.	24.46	16.2	59.5	24.46	16.2	59.5
8.	5.57	17.2	8.26	5.57	17.2	8.26
9.	136.62	50.27	36.1	136.62	50.27	36.1
10.	13.27	9.3	12.95	13.27	9.3	12.95
11.	66.32	180	94.78	66.32	180	94.78
12.	56.96	36.8	30.6	56.96	36.8	30.6
13.	6.6	22.93	5.95	6.6	22.93	5.95
14.	180	180	180	180	180	180
15.	6.8	8.47	16.26	6.8	8.47	16.26
16.	4.41	10.12	22.52	4.41	10.12	22.52
17.	32.76	37.91	180	32.76	37.91	180
18.	17.93	24.37	13.92	17.93	24.37	13.92
19.	24.33	59.08	11.3	24.33	59.08	11.3
20.	30.3	180	180	30.3	180	180
21.	180	180	28.71	180	180	28.71
22.	180	94.56	31.66	180	94.56	180
23.				180	61.6	31.66

Table S2: Times recorded by horses at T10

	Adult horses T10a	Adult horses T10b	Adult horses T10c	Senior horses T10a	Senior horses T10b	Senior horses T10c
1.	2.02	9.81	2.16	180	180	180
2.	10.26	5.36	6.17	96.6	29.52	9.77
3.	24.92	10.27	2.07	35.42	21.76	10.01
4.	13.09	9.49	2.28	28.92	4.11	15.92
5.	9.87	11.51	10.55	41.88	13.72	12.04
6.	4.36	2.89	2.77	3.63	8.64	12.84
7.	5.47	19.1	15.11	31.02	92.78	12.79
8.	8.44	17.13	14.34	28.72	3.37	17.07
9.	31.58	6.72	2.37	41.92	46.05	180
10.	24.54	5.29	15.57	34.37	34.14	19.04
11.	18.38	14.45	9.06	180	180	180
12.	5.47	3.53	2.17	61.11	180	180
13.	3.27	8.31	7.86	5.53	5.66	4.44
14.	27.54	6.22	16.44	180	19.94	180
15.	2.34	17.13	7.69	14.74	180	180
16.	6.39	10.86	2.35	20.77	13.2	34.41
17.	1.92	3.51	2.08	58.2	180	79.1
18.	4.54	2.59	6.22	43.02	33.93	26.37
19.	180	9.86	41.77	9.61	23.52	11.99
20.	39.26	7.03	5.35	180	180	41.86
21.	11.17	5.35	8.32	180	180	180
22.				180	180	180
23.				31.08	53.45	75.28
